# Automatic microdispenser-integrated multiplex enzyme-linked immunosorbent assay device with autonomously driven centrifugal microfluidic system†

**DOI:** 10.1039/d4ra02656j

**Published:** 2024-04-26

**Authors:** Shunya Okamoto, Moeto Nagai, Takayuki Shibata, Yoshiaki Ukita

**Affiliations:** a Toyohashi University of Technology, Department of Mechanical Engineering Japan okamoto@me.tut.ac.jp; b Toyohashi University of Technology, Institute for Research on Next-generation Semiconductor and Sensing Science (IRES^2^) Japan; c University of Yamanashi, Graduate Faculty of Interdisciplinary Research Japan

## Abstract

In this study, we established the control and design theory of an autonomously driven dispenser at a steady rotation speed and proposed a dispenser-integrated multiplex enzyme-linked immunosorbent assay (ELISA) device. In establishing the theory of the dispenser, we estimated the flow rate in the dispenser and the applied pressure onto the passive valves, so that the suitable burst pressure of the valves and flow rate could be designed. The dispenser-integrated multiplex ELISA device has the potential to perform flow control for executing an ELISA of 6 samples/standards per chip or 18 samples/standards per compact disk by just steadily rotating a chip. In the immunoassay evaluation of the device using mouse IgG detection, it was confirmed that the device could assay 5 μL of several standards in just 30 min without nonspecific reactions, and although this system has a high limit of detection (LOD, 63.4–164 pg mL^−1^) it is equal to that of manual assay with a titer plate. The device can be fabricated by transferring the microchannel pattern from a mold without complex assembly or alignment, and it can control the liquid operation by just steadily rotating. Thus, the device system developed will contribute to reducing the cost of fabricating chips and control equipment for ELISA systems. Consequently, a compact, portable, and low-cost ELISA system for point-of-care testing is expected to be realized.

## Abstract 2

A design theory for an autonomously driven dispenser was established. The multiplexed ELISA device was integrated with the dispenser and CLOCK circuits, and successfully prepared a calibration curve of mouse IgG by rotating at a constant speed.

## Graphical abstract

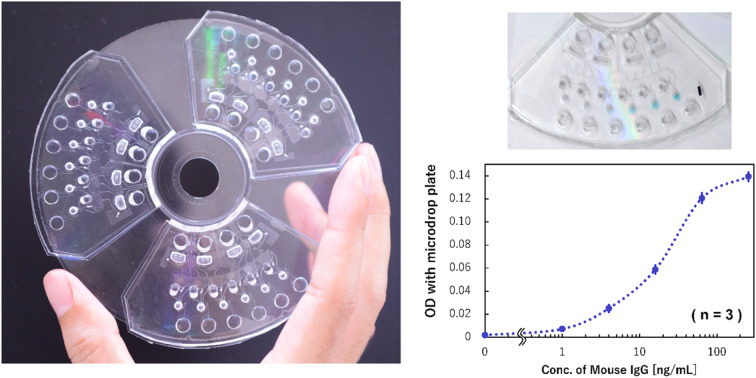

## 1. Introduction

A point-of-care testing (POCT) platform is still a concept, and is one of the methods that could realize a quick diagnostic system from sample collection to diagnosis. It contributes to the prevention of infection spread and the execution of first-aid treatment by giving a rapid diagnosis in pandemics or an emergencies.^1,2^ In addition, as telemedicine becomes widespread with the spread of internet of things, if patients can perform everything from specimen self-collection to analysis by themselves, their quality of life will be improved. Therefore, we hope that the number of test items compatible with POCT can be expanded.^3^ The analytical technique known as an enzyme-linked immunosorbent assay (ELISA) has been used for blood testing. However, because it requires the complicated process of washing reactors, it can be problematic to operate, and it has not yet been completed adapting ELISA to POCT.

Centrifugal microfluidic devices have the advantage of automation of chemical analysis using a few samples and reagents because they can predominantly control liquid behavior against unstable forces, such as surface tension and capillary force, by applying a high centrifugal acceleration greater than 100 × *g*.^4,5^ Several assay applications using a centrifugal microdevice have been explored, *e.g.*, the polymerase chain reaction,^6–8^ loop-mediated isothermal amplification,^9–11^ ELISA,^12–14^ and so on.^15–17^ Among these techniques, ELISA presents the most challenges for POCT applications because it needs to compare the assay results of samples with the calibration curve of the quantitation, and the preparation of both the sample assay and calibration curve need to be performed under the same environmental conditions. In other words, to adapt an ELISA system to POCT, the system is required to assay only the sample on the site under the same conditions used to prepare the calibration curve by giving some functions to the system, or it assays the sample and prepares the calibration curve synchronously on-site. However, the former may be inadequate for on-site diagnosis as the system may become larger as well requiring guarantees and maintenance from various perspectives, including environmental conditions, such as temperature, and reagent storage. Meanwhile, a centrifugal microfluidic device can easily contribute to parallel processing, and some examples of systems that execute some assays on a one chip-sharpened disk have been proposed.^18,19^ Nevertheless, these systems put a burden on the user because they require the user to load some reagents into several reservoirs.^20,21^ This problem can be solved by integrating a microfluidic control technique. Typical examples are dispensers,^22–24^ which can inject into some reservoirs from an upstream reservoir, and they play a role in increasing the number of samples/standards without increasing the user's burden. However, a dispenser that simply drives and has a low cost is required that can be adapted for use in the POCT.

In previously reported conventional dispensers on centrifugal microfluidic devices, a method in which liquids are metered on some reservoirs placed upstream of the destination reservoir, and air is forcibly replaced in the injecting reservoir with liquid by increasing the rotation speed (centrifugal force) has become mainstream.^25–28^ This method^29^ is easy to implement but does not incorporate an additional process, such as liquid replacement in the used reservoirs.^30^

Moreover, a method using hydrophobic valves in place of forcibly replacing air^31^ and a centrifugo-pneumatic multi-liquid aliquoting method^32^ have been proposed. However, these dispensing methods require switching rotation speeds. Switching rotation speeds requires intricate setups or additional equipment and poses a risk of unstable liquid behaviors due to Euler's forces. Therefore, we think a centrifugal microfluidic device for POCT must be driven at a steady rotation speed for simplicity and stability because this will cost issues and centrifugal acceleration can be applied uniformly and dominantly in the same direction to the unstable surface tension that causes errors. Thus, we reported the concept of an autonomous driving dispenser at a steady rotation speed and confirmed that the dispensing volume deviations are very small with a coefficient of variation of less than 5% (original paper written in Japanese).^33^

**Fig. 1:**
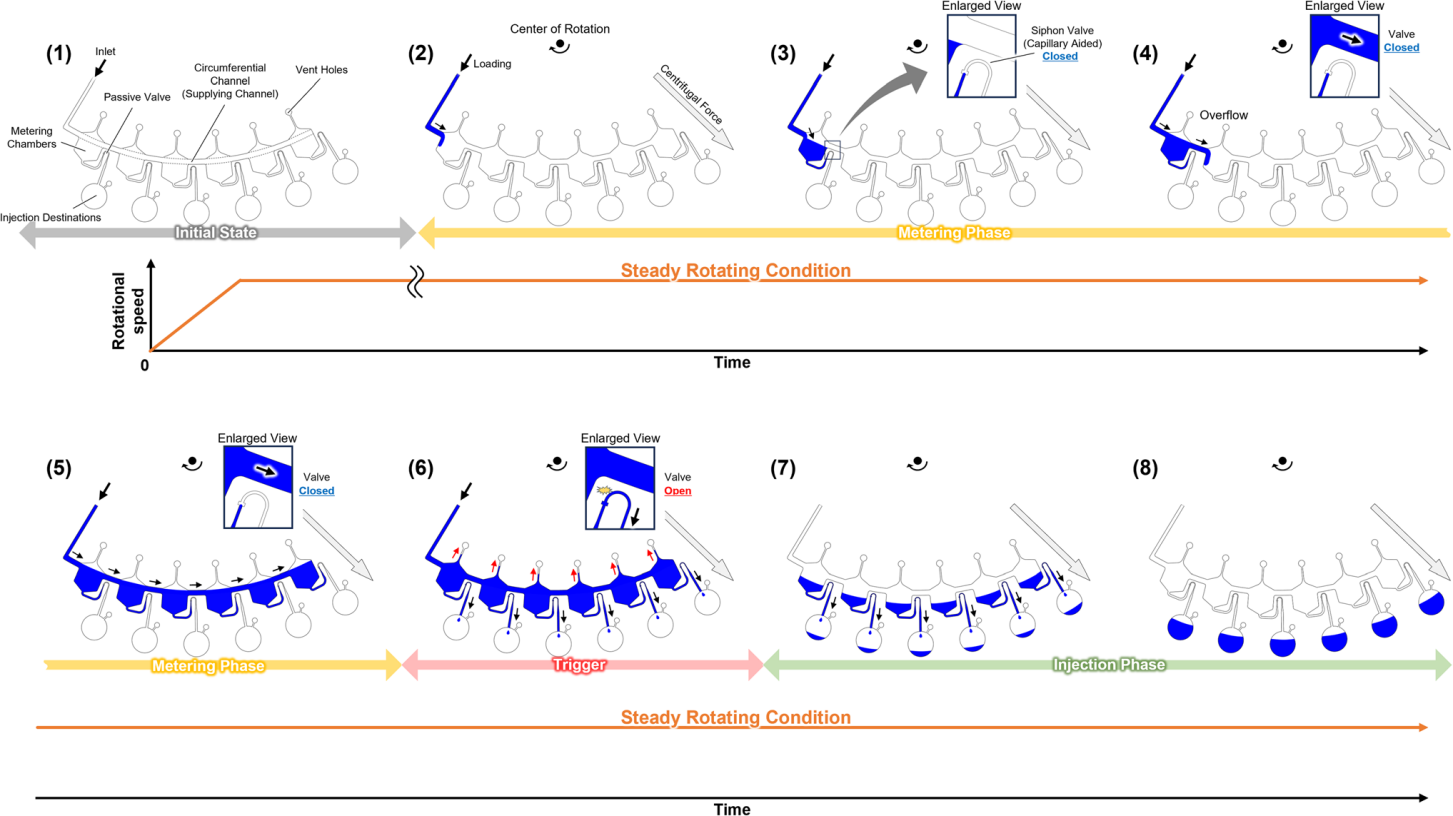
The operation principles of the autonomously driven dispenser. (1) Initial state. (2)–(5) Metering phase; the valves are closed. The liquid is loaded from the upstream (CLOCK circuit) and metered as the metering chambers overflow. (6) The valves are triggered and opened by the increasing water levels. (7) and (8) Injection phase, the valves are closed when the injections are performed so that the dispenser can dispense repeatedly.

Fig. 1 shows the operating principle of the autonomous driving dispenser. The dispenser comprises a set of metering chambers and valves, which are arranged according to the number of target chambers (dispensing destinations). Each metering chamber is connected by a circumferential channel (supply channel). The loaded liquid is simultaneously injected into all the target chambers after metering in the corresponding metering chambers *via* the supplying channels. The valves are passive valves, such as capillary valves or three-dimensionally crossed siphon valves, and are connected with the supply channels. Also, according to the design specifications, a burst pressure is used that can withstand the pressure experienced during the metering phase. Rapid water level changes in the dispenser are employed to trigger injection after the metering phase. In particular, by reducing the volume of the upper part of the metering chambers, the head pressure can be applied rapidly to all valves without changing the loading flow rate. Therefore, the pressure required for opening the passive valve, *P*_valve.burst_, the pressure applied to the valve during the metering phase, *P*_applied_, and the pressure applied to each valve at the rising water level after the metering phase, *P*_head_ must satisfy the following inequality:

1 *P*_head_ > *P*_valve.burst_ > *P*_applied_

However, in previous research, *P*_applied_ was not clearly estimated during the metering phase.

Therefore, in this study, we established the mechanism and design theory of the dispenser. In addition, we designed and demonstrated multiplex ELISA devices that can be driven at a steady rotation speed by integrating a CLOCK circuit^34^ and the autonomous driving dispenser.

## 2. Materials and methods

### 2.1 Fabrication of centrifugal microfluidic chips and observation methods

Microfluidic chips were fabricated using general photolithography and soft lithography methods.^35^ First, negative photoresists (SU-8 3050 or 2100, KAYAKU Advanced Materials, Inc.) were spin-coated onto 10.2 cm silicon wafers to obtain the target thicknesses. The spin-coated wafers were heated at 95 °C for pre-exposure baking. The wafers were exposed to ultraviolet light and then covered with the photomask to obtain patterned microchannels. Next, the post-exposure baking was performed at 65 °C and 95 °C. All the molds used to fabricate the microfluidic chips were prepared using a multilayered photolithography process, by being additionally spin-coated, pre-exposure baked, exposed to ultraviolet light, and post-exposure baked several heights of microchannels were obtained. Finally, patterned microchannels were developed using an SU-8 developer, and the molds were obtained by rinsing them with isopropanol. These patterned photoresists are mainly used to form microchannels and a dispenser. The secondary reservoirs of the assay devices were shaped by placing PMMA parts cut to a designed size on the photoresists with double-sided tapes. The reaction chambers were formed by mounting waxes (Ferris File-A-Wax, Freeman Manufacturing & Supply, Avon, OH, USA) onto the photoresists using a reflow process.^36^ A PDMS monomer and curing reagent (SILPOT 184, Dow Corning Toray Co., Ltd., Japan) were mixed at a ratio of 10 : 1, and then the mixture was applied to the molds. To obtain a certain thickness of the PDMS chips, the edges of the molds were covered with silicone rubber as a spacer, and PET sheets and glass substrates were placed on the surfaces during curing. The PDMS chips were cured by heating at 75 °C for 90 min, and after peeling them off from the molds, the cured PDMS chips were heated additionally at 200 °C for 30 min to increase their hardness. Inlet reservoirs, waste chambers, and venting holes of the chips were fabricated using a punching process. A 1 mm-thick PDMS sheet was bonded to the channel side of the chips using a plasma process to obtain the microchannels. The PDMS chips were mounted on compact disk substrates with double-sided tapes. A clear adhesive tape (640 PF D-50, NICHIBAN Co., Ltd., Japan) was placed on the chip surfaces to shut the reservoirs fabricated by punching. The parts closing the ports for loading reagents and the venting ports were then cut out using a knife. Moreover, to immobilize the capture antibodies before the assay and to collect products from the reaction chambers after the reaction, holes were made in the chambers using an 18 G needle.

The microfluidic chips were rotated with custom-made centrifuge equipment,^37^ and to observe flow behavior in real time, a stroboscope^38^ was used. The flow rate was measured with an image analyzer using captured images. Further information, and more detailed documents and schematic images for the multiplex ELISA device are provided in the ESI.†

### 2.2 Preparation of reagents

To test the dispenser, a 0.1% (w/v) safranine (196-00032, Wako Pure Chemical Industries, Ltd., Japan) aqueous solution was used as the working fluid. For the ELISA, Dulbecco's phosphate-buffered saline (DPBS) was prepared using a general method. Bovine serum albumin (BSA, A7030-50G, Sigma-Aldrich Japan Co., LLC, Japan) was dissolved in DPBS at a concentration of 1% (w/w) and used as a blocking buffer, sample/standard diluent, and conjugate diluent. Tween 20 (167-11515, Wako Pure Chemical Industries, Ltd., Japan) was added to DPBS at a concentration of 0.05% (v/v) and used as the washing solution. Phosphoric acid was diluted to 1 M with ultrapure water and used as a reaction stopper for a 3,3′,5,5-tetramethylbenzidine (TMB) peroxidase substrate (5120-0053, SeraCare Life Sciences, Inc.). In a demonstration of flow control of a multiplex ELISA device, 1% (w/v) safranine was dissolved in the sample/standard diluent, and 1 mM fluorescein (F6377-100G, Sigma-Aldrich Japan Co. LLC, Japan) was added to the wash solution for liquid visualization. The TMB was reacted with a small amount of horseradish peroxidase (HRP) and was colored.

### 2.3 On-chip ELISA preparation

We used a mouse IgG detection system on a multiplex ELISA device using the commercially available ELISA Quantitation Kit (E80-129, Bethyl Laboratories, Inc.). To immobilize the capture antibodies onto the reaction chambers, 7 μL of goat anti-mouse antibody (#31093, Invitrogen, USA) diluted 100-fold in DPBS was applied to the chambers, and the mixture was incubated for 2 h at room temperature. The reaction chambers were washed three times with the washing solution, vacuum dried overnight, and then used for the assay without a blocking process using BSA. The washing solution (50 μL) was put into three primary reservoirs for the washing control circuits, the TMB solution (50 μL) was put into the primary reservoirs, and the standards (5 μL) were put into the sample/standard inlet reservoirs. The standards were prepared using a mixture of the prereacted mouse IgG (#31903, Invitrogen, USA) with HRP-labeled goat anti-mouse IgG (074-1806, Kirkegaard & Perry Laboratories Inc., USA) for 20 min in micro tubes. The reagents and standard loaded devices were steadily rotated at 1620 rpm (27 Hz) for 25 min. The acceleration and de-acceleration for approaching the target rotation speed and stopping were both set to 300 rpm s^−1^. After stopping the rotation, 5 μL of the product kept in each reaction chamber was collected and reacted with a uniform volume of the reaction stop solution. The optical densities (ODs) were then measured using a microplate reader and microdrop plate (Multiskan GO Microplate Spectrophotometer, Thermo Fisher Scientific Inc., USA).

## 3. Results and discussion

### 3.1 Estimation of flow rate and applied pressure on the dispenser

**Fig. 2:**
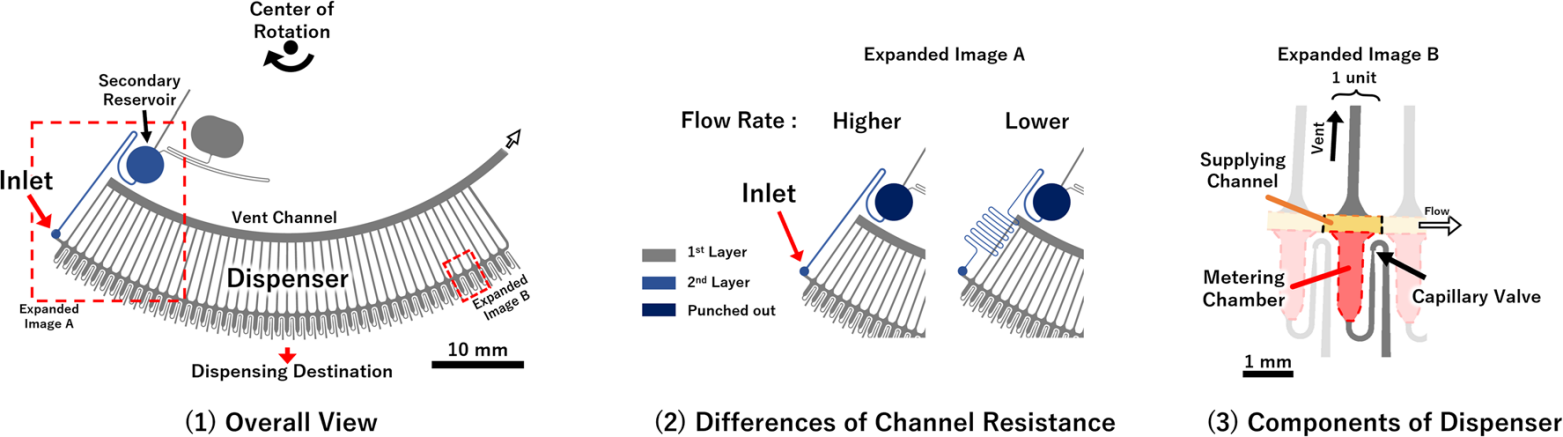
Detailed structure of the test dispenser device. The loading flow rate into the dispensers was controlled by adjusting the resistance of the channel between the secondary reservoir and the dispenser inlet. To reduce the differences in the shapes of other parts, such as the channels and chambers, they were fabricated with the same mold, and the device was comprised of two-layered structures created by assembling the PDMS chips patterned on the microchannels.

To experimentally investigate *P*_applied_ on each valve in the metering phase, test devices were fabricated to visualize the pressure on the valve using the burst of the capillary valves as an indicator. Two types of devices were fabricated, with different liquid loading flow rates, into the dispenser (Fig. 2). In addition, the rotation speed of the devices was also used as a parameter. The test devices were designed as autonomous driving dispensers and comprised circumferential direction channels (supplying the channel), metering parts, and vent valves, which were connected to 45 units. Note that, in actual use of the dispenser, the flow rate of liquid into the dispenser must be higher than the sum of the outflow flow rates (injection flow rates). However, in whichever case the multiple valves would burst simultaneously, and it was difficult to observe the phenomenon, so the outflow flow rate was designed to be high for the test devices.

**Fig. 3:**
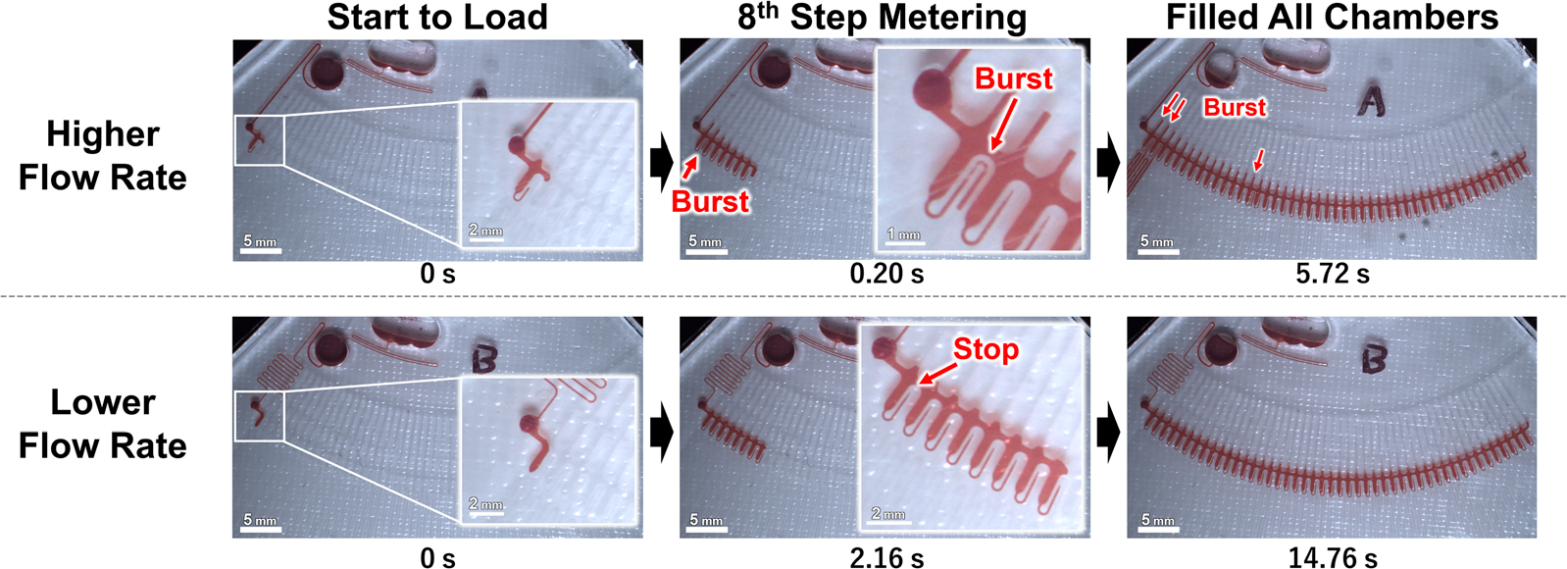
The observed flow behavior of each device at 1500 rpm.

Fig. 3 and the ESI Movies† show the observed flow results at 1500 rpm. At the higher flow rate, when the liquid was flowing to the eighth metering chamber, the capillary valve of the first metering chamber from the left, burst. Meanwhile, at the lower flow rate, neither of the capillary valves burst until all metering chambers were filled. The burst pressures of the capillary valves of the test devices were calculated to be 3.61 ± 0.23 kPa (*n* = 18) from the measured dimension of the valve structure according to a previous study.^24^ Thus, to burst a valve means that pressure of more than 3.61 kPa was applied to the valve.

When the liquid is pumped into a microchannel by pressure, the largest pressure is applied to the most upstream microchannel, and the greater the resistance (longer or narrower microchannels) or the higher the flow rate, the larger the applied pressure. We thought that the pressure was applied to the valves, and this was a pressure drop. In particular, this pressure drop *P*_drop_ can be expressed as follows:^39^2
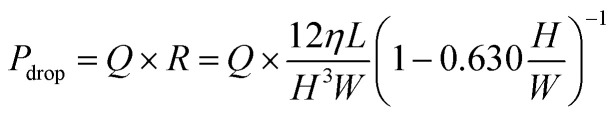
where *Q* denotes the flow rate, and *R* denotes the microchannel resistance. Moreover, the resistance in a rectangular microchannel (width *W*, height *H*, and length, *L*) can be theoretically described by eqn (2), where *η* denotes the liquid dynamic viscosity. Thus, we thought that, in the dispensers, estimating the flow rate was also required to simulate the applied pressure to the valves. Therefore, we estimated the flow rate in a dispenser, especially in the supply channel.

In a centrifugal microfluidic device, the applied pressure *P*_centrifugal_ for the driving force is generally described by eqn (3),^18^ and is determined by the microchannel shape, physical properties, and liquid levels. In addition, the flow rate can be calculated using eqn (4):^40^3
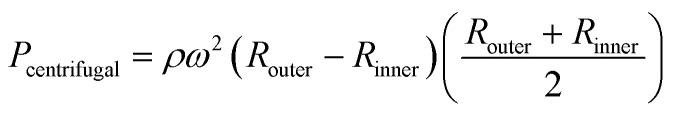
4
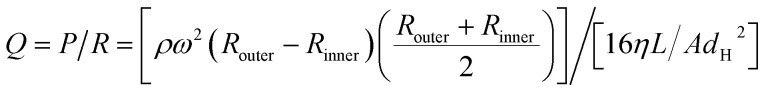
where *ρ* denotes the working liquid density, *ω* denotes the angular velocity, *A* and *d*_H_ denote the cross-sectional area and equivalent diameter (=4*HW*/(*H* + *W*)) of the microchannel, respectively, and *R*_outer_ and *R*_inner_ denote the distance to the leading meniscus or upstream meniscus from the rotation center. In eqn (4), the resistance could be estimated by measuring the microchannel dimensions and the working liquid properties. Therefore, the parameters required to identify the applied pressure are the water levels, namely, the position of the meniscuses.

**Fig. 4:**
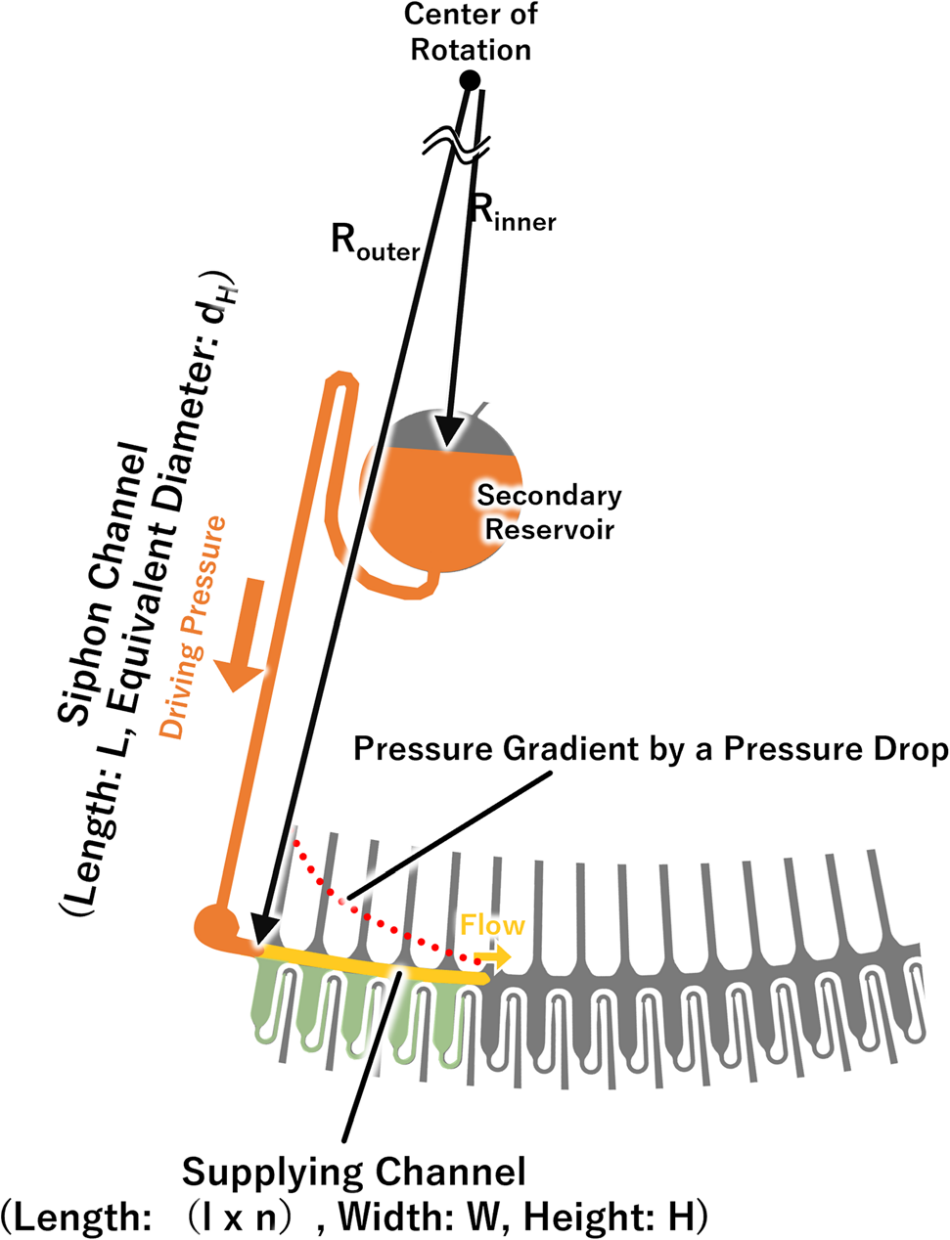
Schematic of the major parameters used for estimating flow rate and applied pressure.

The positions were generally set to the two meniscuses, one upstream and one downstream. However, when the flow rates were simulated in a dispenser according to these conditions, the results did not correspond to the experimental results. In the simulation, only the water head pressure from the secondary reservoir to the inlet of the dispenser was considered to be the driving pressure, although this was thought to be because it did not reflect the increasing pressure at the inlet (outlet of the siphon channel) due to the increase in the pressure drop in the supply channel. Therefore, by considering the water head pressure as a positive pressure applied to the pumping direction and considering the pressure equivalent to the pressure drop given in eqn (2) as the negative pressure applied to prevent pumping, a new flow equation was defined as follows:5
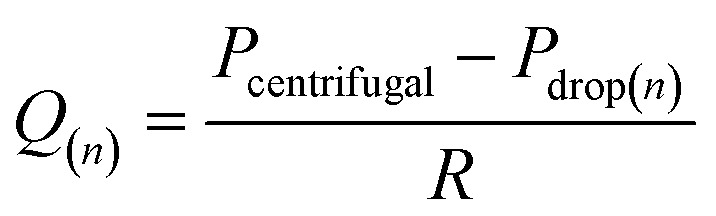
6
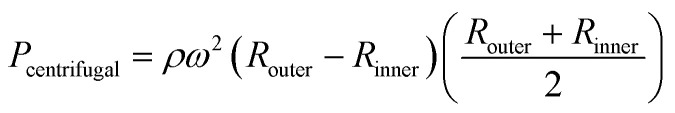
7
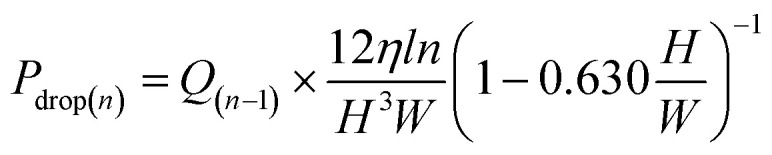
where the channel length *L* in eqn (2) is a function that increases with the number of dispensations (number of meterings) *n*, so the length was defined as “*l* × *n*” in eqn (7), where *l* denotes the length of the supply channel per unit of metering chamber and *R*_outer_ and *R*_inner_ are defined as shown in Fig. 4.

**Fig. 5:**
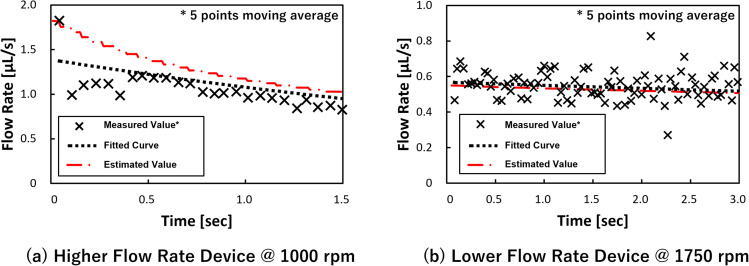
The measured flow rate compared with the estimated values.

Consequently, the flow rate was re-estimated using these equations, and the values were compared with the experimental values, and the results are shown in Fig. 5. The experimental values represent the two conditions in which sufficient measurement time was obtained for each device. Although there are some small and large differences, it was decided that the flow rate had been adequately estimated. In addition, during the metering phase, the water level of the secondary reservoir, which corresponds to *R*_inner_ in eqn (6), did not change significantly because the volume of the secondary reservoir (18.8 μL) was sufficiently large when compared with the volume of the entire dispenser (4.15 μL). This means that the water head pressure applied to the liquid in the dispenser from the secondary reservoir did not change. Meanwhile, the estimated flow rate decreased. Therefore, it is considered that the equation can be used to estimate the decrease in flow rate due to the increased pressure drop. In particular, the experimental values periodically changed from large to small because the flow rate was momentarily different depending on whether the leading meniscus moved in the circumferential direction or in the metering chamber direction (radial direction) in the supply channel-section, and this is considered to be the reason for the large and small values that appeared in relation to the taking image cycle (one piece per revolution).

**Fig. 6:**
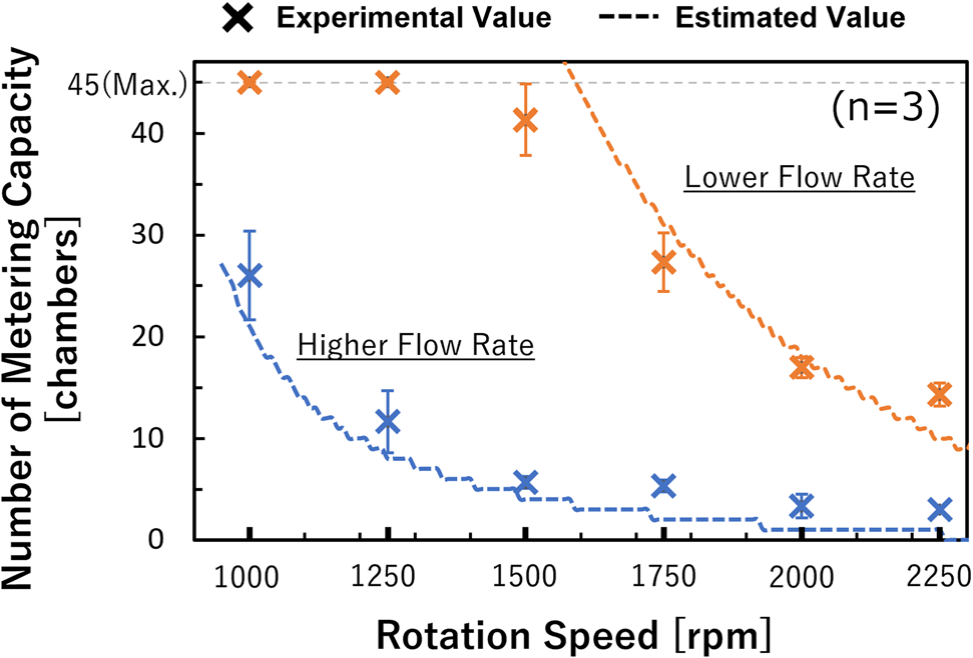
The number of metering capacity chambers for each rotation speed and device.

From the experimental work described above, it was possible to simulate the flow rate in the dispensing mechanism. We estimated the pressure applied to the valve and the number of chambers available for metering (metering capacity) using eqn (2) and (7). The accuracy of the estimation was evaluated using the rotation speed as a parameter. The results are shown in Fig. 6, and the results for both types of devices were generally consistent with the theoretical values. For the lower flow rate, the experimental metering capacity tended to be smaller than the theoretical value in the high rotation speed range. This is thought to be because the metering takes time with a low flow rate, whereas the wettability near the capillary valve changes, thus, the burst pressure of the capillary valve is reduced. In particular, the water head pressure caused by the height difference between the supply channel and the capillary valve were considered in this theoretical value estimation, in addition to the pressure drop in eqn (2). Therefore, the pressure applied to the most upstream valve during the metering phase can be expressed as:8
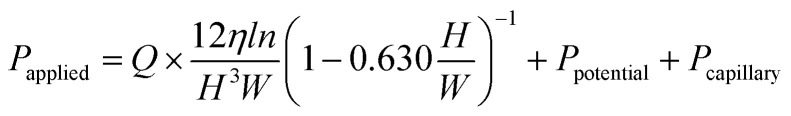
where *n* denotes the number of dispensations, *P*_potential_ and *P*_capillary_ denote the water head pressure by the position on the valve and the Laplace pressure, respectively, and the flow rate *Q* can be estimated using eqn (5). Moreover, it is considered that the burst pressure of the valve needs to be designed to satisfy eqn (1) and (8).

### 3.2 Evaluation of a dispenser-integrated multiplex automatic ELISA device

#### 3.2.1 Demonstration of flow control

**Fig. 7:**
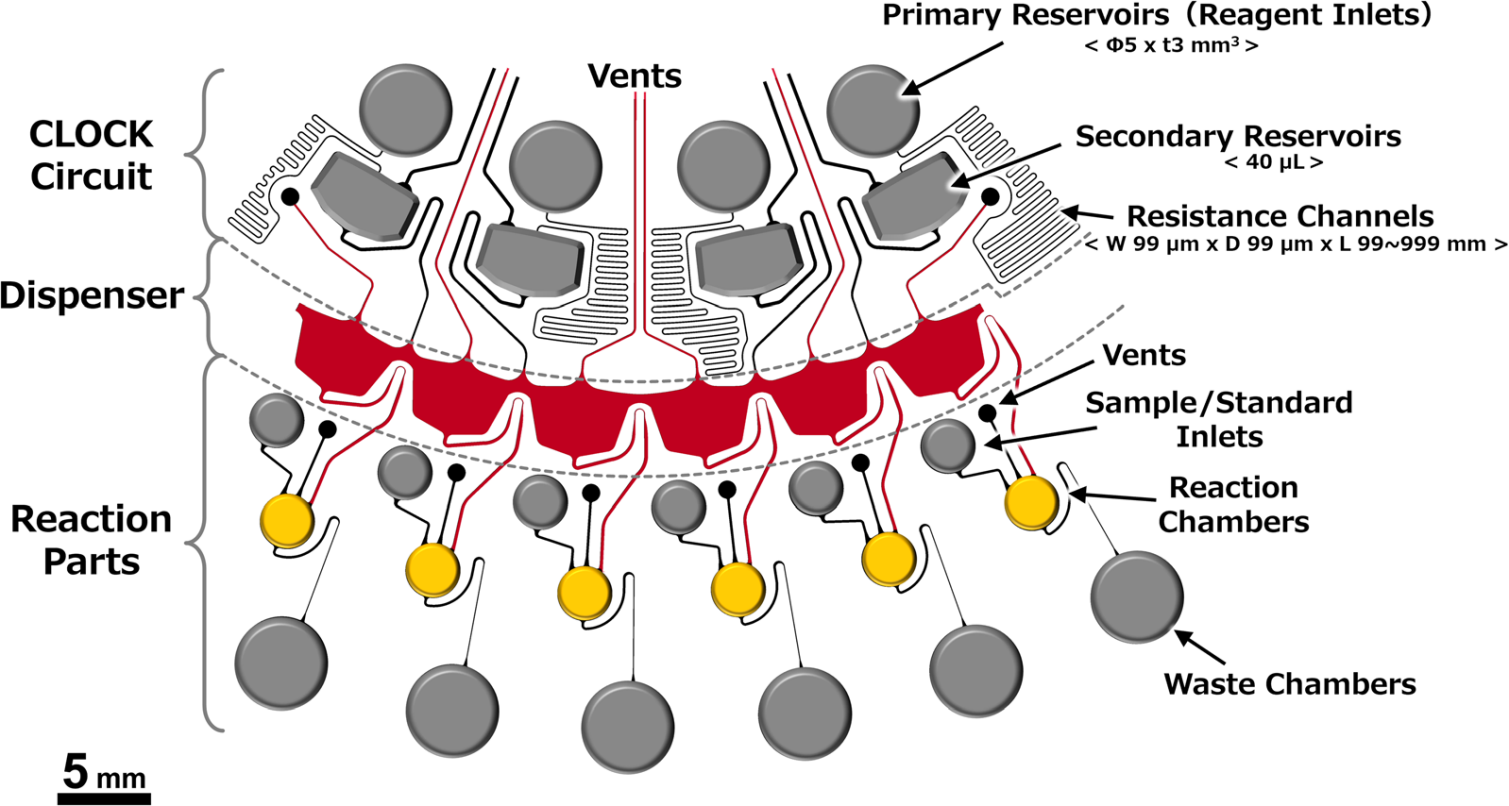
A schematic of the structure of the dispenser-integrated multiplex ELISA device. This device consists of a CLOCK circuit (sequencer), dispenser, and reaction parts. The washing solution and TMB substrate are applied to each primary reservoir, and the samples/standards are applied to each inlet reservoir located upstream of each reaction chamber. The primary reservoirs, sample/standard inlets, and waste chambers were fabricated by punching out the PDMS chips after soft lithography, and the shapes of the reaction chambers and dispenser were patterned onto the molds using a reflow process and photolithography.

**Fig. 8:**
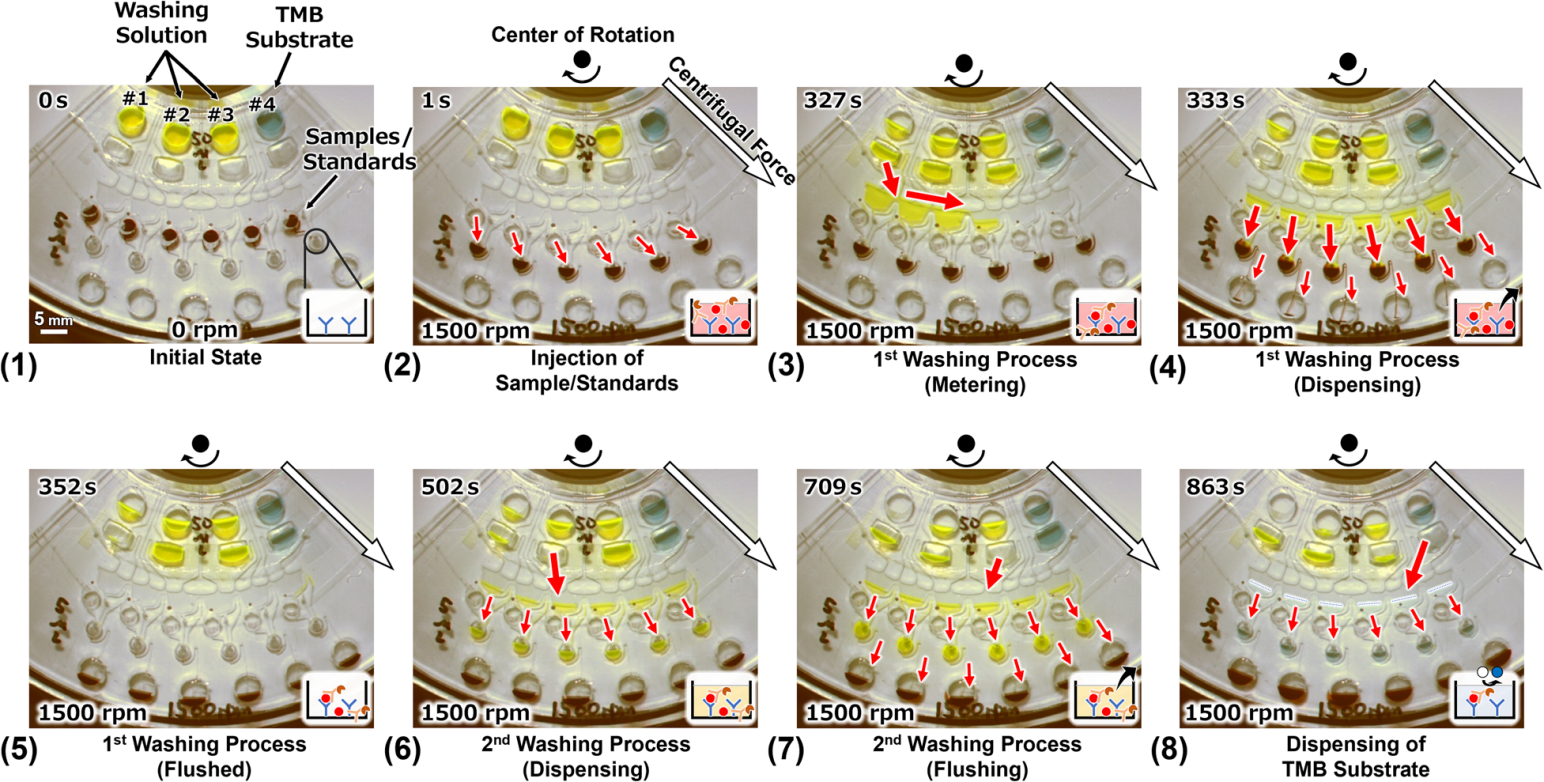
The results of the flow control demonstration for ELISA, and the images show the reaction chambers. (1) Initial state: 50 μL of the colored washing solution (yellow) and the color developed TMB (blue) were applied to each of primary reservoirs #1–#4, and 5 μL of the colored sample/standard diluent (red) was applied to each inlet reservoir. (2) The start of rotation (1500 rpm) and flow. (3)–(5) First washing process. (6) and (7) The second washing process. (8) Dispensing the TMB and holding it in the reaction chambers.

Multiplex ELISA devices integrated with a dispenser that can dispense into six reaction chambers were designed and fabricated based on the above results. The CLOCK circuits^34^ were placed upstream of the dispensers (Fig. 7), and they played a role in controlling the injection timing of the washing solution and the TMB substrate (the operating principle is described in the ESI†). Firstly, we demonstrated a device with colored reagents. The observed behaviors are shown in Fig. 8, and the Movie is found in the ESI.†

Before rotation, the loaded liquids were kept in each reservoir (Fig. 8(1)). When the rotation was started, each liquid started to flow, and the samples/standards were injected and kept in each reaction chamber (Fig. 8(2)). In the actual assay, the capture antibodies were previously immobilized onto the reaction chambers and the antibody–antigen reactions were initiated from this operation. The washing solution and the TMB substrate were accumulated in each secondary reservoir *via* the resistance channels. The resistance channels and the secondary reservoirs were components of the CLOCK circuits, which were sequenced to inject each reagent into the dispenser. Exactly 327 s after the start of the rotation, the washing solution was injected into the dispenser from secondary reservoir #1 (Fig. 8(3)). The solution was then dispensed into the six reaction chambers simultaneously in the metering phase. The injected solution filled the reaction chambers and then the triggered the siphon valve opening downstream of the chambers. The solution drained out into the waste chamber and the samples/standards remained in the reaction chambers (Fig. 8(4)). Then, the reaction chambers temporarily became empty (Fig. 8(5)). Next the antigen–antibody reaction was started, which is the first washing process. Subsequently, 502 s after the start of rotation, the washing solution was dispensed into each reaction chamber *via* the dispenser from the secondary reservoir #2, and the washing solution remained in the reaction chambers (Fig. 8(6)) because the dispensing volume of the dispenser was designed so that it could fill the reaction chambers using two dispensing operations. Moreover, 709 s after the start of rotation, the washing solution was dispensed into each reaction chamber, which was filled with the washing solution. Then, the siphon valve downstream of the chambers was opened, and all the liquids in the chambers were drained out into the waste chamber. This was the second washing process. Finally, the TMB substrate was dispensed into each reaction chamber and left in there. This is a color developing reaction with the HRP conjugates in the actual assay. Although the device was rotated for 25 min in total, the TMB substrate remained in all reaction chambers. Thus, it was demonstrated that the device can be used to perform ELISA on six samples/standards simultaneously in steady rotation.

#### 3.2.2 Demonstration and evaluation of the immunoassay with the device system

**Fig. 9:**
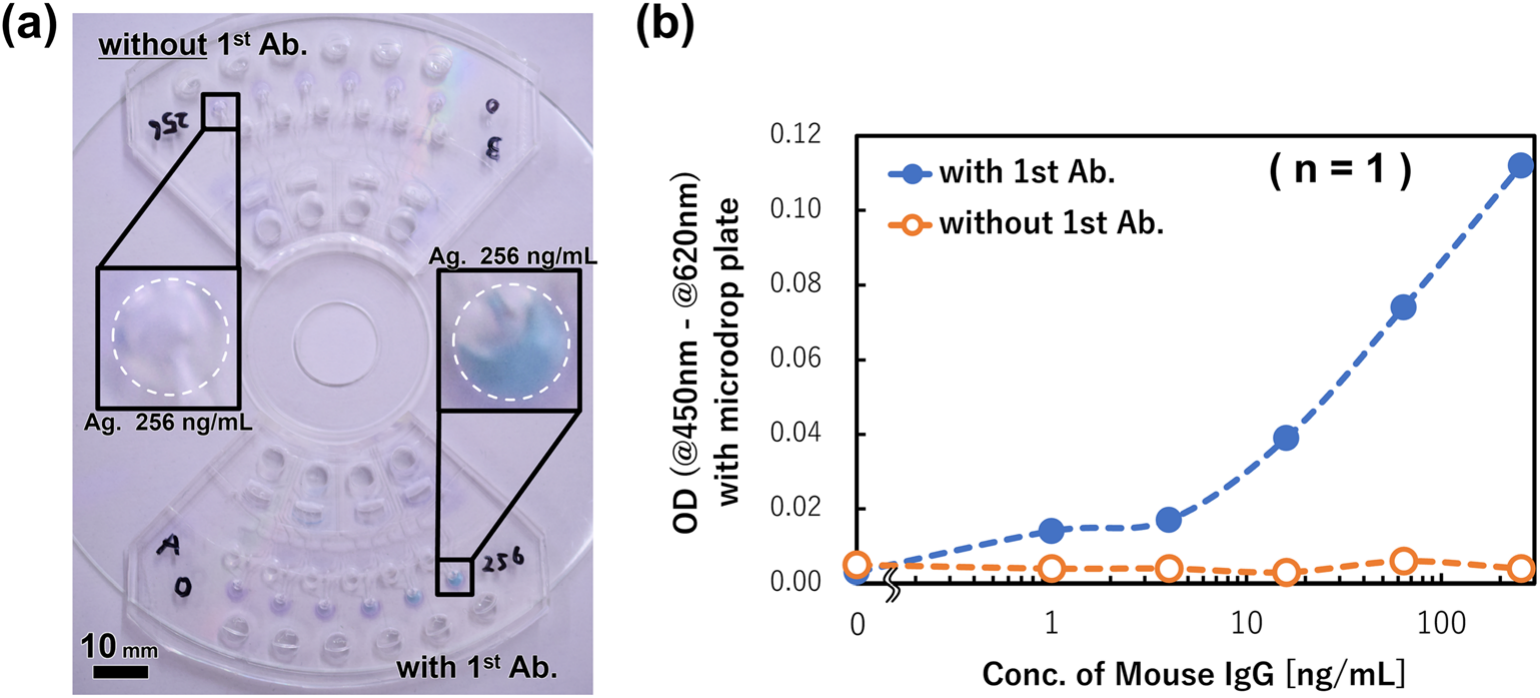
Results of immunoassay demonstration using the devices. (a) Images of the devices after the TMB reactions: the assay was executed without any capture antibody (1^st^ antibody) in the upside device; in the downside device, it was executed with 1^st^ antibodies. (b) The calibration curve obtained with the devices shown in (a).

First, we performed an ELISA using 12 standards with the two devices set on a compact disk (CD), however, the capture antibody was immobilized onto the reaction chambers in only one device. The concentrations of six antigens (mouse IgG) were within 0–256 ng mL^−1^. Fig. 9 shows the image after the rotation and the measured ODs of the products that were collected from the reaction chambers and reacted with 1 M phosphoric acid. Using the capture antibody, the dose–response of the antigen concentration was obtained. Meanwhile, without using the capture antibody, only a background level of the signal was observed at any antigen concentration. From these results, it is thought that the coloration of the TMB obtained indicates that the antigen–antibody reaction had successfully occurred without nonspecific reactions in the reaction chambers. In other words, it is considered that the device is effective as a detection system for mouse IgG.

**Fig. 10:**
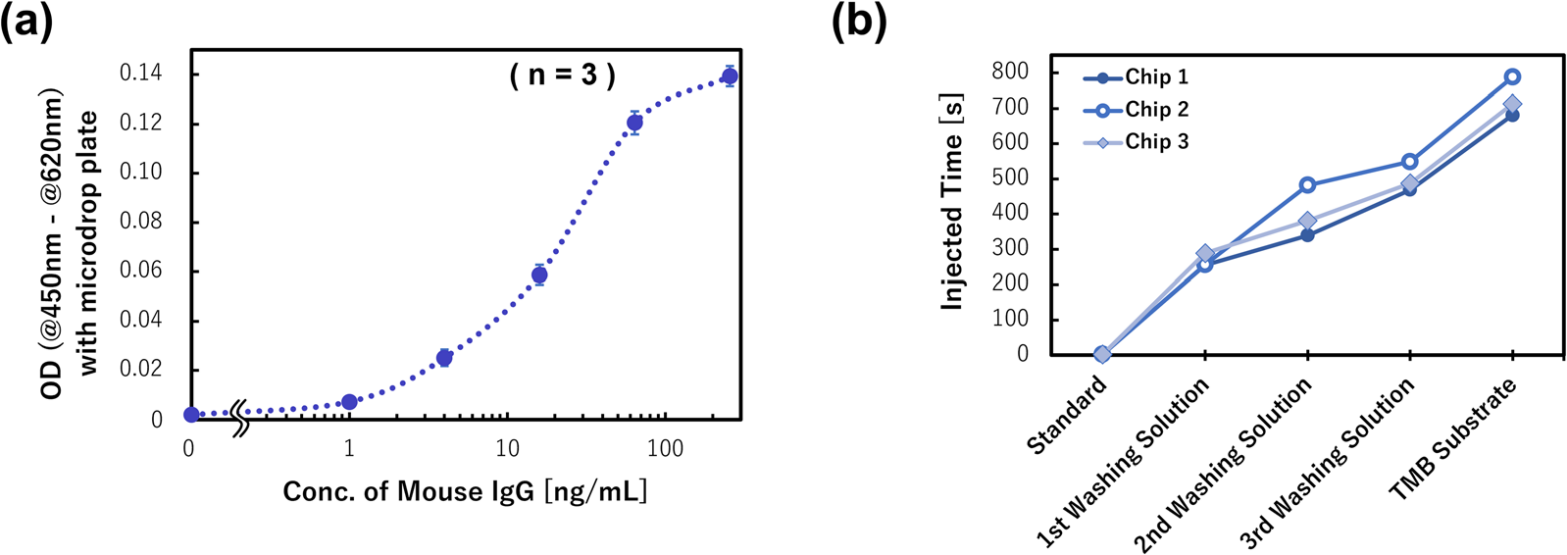
The prepared calibration curve of the mouse IgG detection system and the timing control performance of the CLOCK circuit.

Finally, we prepared the calibration curve for the mouse IgG detection using three devices for the six antigen (mouse IgG) concentrations within 0–256 ng mL^−1^ (*n* = 3), and the results are shown in Fig. 10. The measured ODs of products after rotation were obtained from the dose–response curve of the antigen concentration, and it was confirmed that the sigmoid shaped calibration curve was successfully prepared. The limit of detection (LOD = 3.3 × SD/slope) was calculated to be 164 pg mL^−1^ in this assay, and the champion data was 63.4 pg mL^−1^. This detection ability is equal to that of a manual assay with a 96 well titer plate. However, compared with the manual assay, the devices realized the same detection ability and the antigen–antibody reaction time was reduced from 60 min to 5 min. A previously reported single assay device^34^ also exhibited the same reaction time, although the multiplex ELISA device improved the detection sensitivity by one order of magnitude. This is because by reducing the sample/standard volume (*e.g.*, to one-tenth of the manual assay and one-sixth of the single assay device), the specific surface area was increased and the reaction efficiency improved. In addition, the multiplex ELISA device can align the reaction time on the same device, so that the differences in the conditions between the various reaction chambers are small. It is also thought to be one of the factors for improving the detection sensitivity. Therefore, although three chips mounted on a CD substrate were used to prepare the calibration curve due to the limitation of the chip fabrication in this study, if a device that can assay 18 samples/standards on one chip is fabricated, it is expected to realize a higher performance. This can be realized using the developed dispenser and multiplex ELISA device concept.

## 4. Conclusions

In this study, the design conditions of an autonomously driven dispenser was established, and a multiplex ELISA device integrated with the dispensers is proposed. In the dispenser operation theory, we confirmed that the flow rate in the supply channel and applied pressure onto each valve can be calculated without advanced fluid control simulation software. As a result, it can be said that the dispenser's design criteria have been established.

In the demonstration of a multiplex ELISA device with colored reagents, we confirmed that the device can perform the flow control required to execute an ELISA of six samples/standards on a chip with just steady rotation. This system can assay 18 samples/standards per CD substrate, which is more than those of recently reported ELISA devices.^20,21,41^ In addition, we demonstrated the use of an ELISA for mouse IgG detection using the device, and the dose–response of the mouse IgG concentration within the range of 0–256 ng mL^−1^ was successfully obtained. The device was validated as a mouse IgG detection system because no other signals were observed besides the capture antibodies.

The proposed device system can simultaneously assay samples and standards, including the generation of the calibration curve, in less than 30 min while maintaining a detection sensitivity equal to a manual assay with titer plates. The required sample/standard volume is just 5 μL, so samples can be collected from a fingertip using a lancet. In addition, this device system can be fabricated using one time soft lithography from a single mold without a complex assembly or additional treatment and processing, indicating that it can be fabricated using a process suitable for large-scale production, such as injection molding, as in the case of a previously proposed chip.^42^ Moreover, we have already developed a centrifugal microfluidic device which can extract plasma from whole blood with a steady rotation speed.^43^ Because both have the concept of operating in steady rotation, we think that they can be easily integrated. Overall, because the device system is convenient for performing immunoassays and reduces the analysis costs, it is anticipated that it will be implemented as soon as possible in a POCT blood testing system that is compatible with immunoanalytical equipment.

## Conflicts of interest

There are no conflicts to declare.

## Supplementary Material

RA-014-D4RA02656J-s001

RA-014-D4RA02656J-s002

RA-014-D4RA02656J-s003

RA-014-D4RA02656J-s004
