# Supplementary material for: Automatic microdispenser-integrated multiplex enzyme-linked immunosorbent assay device with autonomously driven centrifugal microfluidic system
Source: RSC Adv. 2024 Apr 26;14(20):13827–36. doi: 10.1039/d4ra02656j (PMC11047056; doi:10.1039/d4ra02656j)
Supplement: RA-014-D4RA02656J-s001 [file RA-014-D4RA02656J-s001.pdf]

Electronic Supplementary Material (ESI) for RSC Advances.
This journal is © The Royal Society of Chemistry 2024

Supplementary information

Image analysis for measuring flow rate in a dispenser

After binarizing the captured images and identifying the liquid's portions, the difference between the number of pixels and the period between consecutive captures was used to calculate the increase or decrease in the number of pixels (corresponding to the liquid's area) per unit of time. Additionally, the liquid's height and area were multiplied to determine the flow rate.

By identifying the value at which the area difference becomes the smallest value when compared to the liquid area of the captured image, the threshold value of binarization was established using the CAD data used for the photo mask. Notably, the captured image had liquid in several regions, making it possible to precisely determine the meniscus's location. Moreover, the conversion between pixels and actual distance was achieved by comparison. The liquid's height was calculated by averaging several measurements of the microchannels' depth.

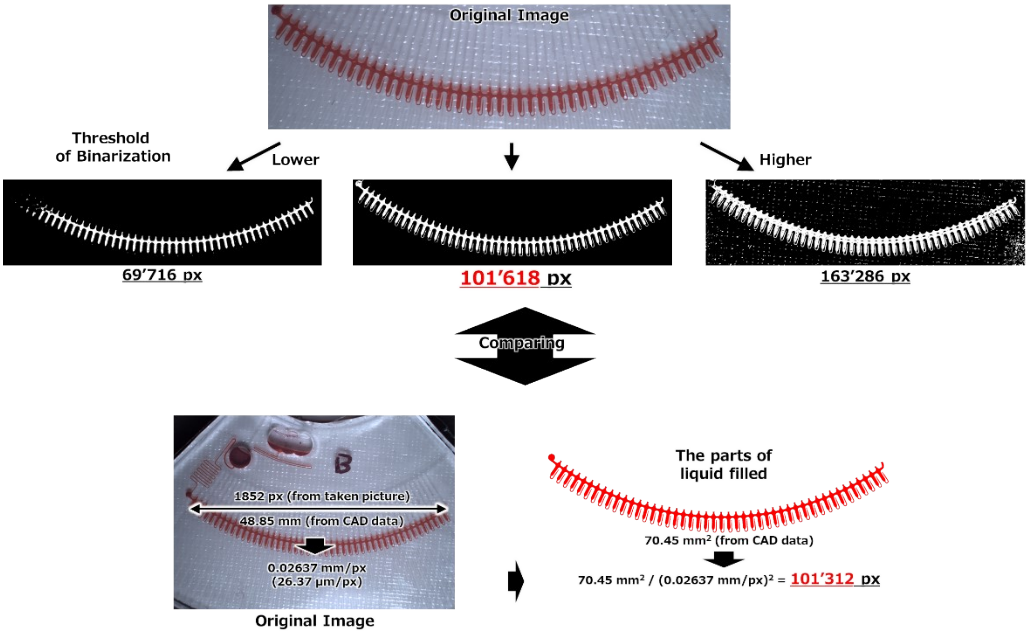

Fig. S1 Methods for conversion between pixel and actual distance and area in image analysis

Operation principle of CLOCK circuit

CLOCK circuits played roles of control for injection timing of reagents (washing solution, and TMB substrate) into the dispenser. The principle of operation is shown below.

The channel of each reagent was composed of a primary reservoir, resistance channel, secondary reservoir, and siphon valve from upstream. A reagent which was controlled injection timing, was applied into the primary reservoir. By stating rotation, the reagents started to flow into the secondary reservoir through the resistance channel. The reagent was kept in the reservoir because the siphon valve was placed downstream the secondary reservoir. Then, when the reservoir was filled, the siphon valve burst, and the reagent was injected into the dispenser. Therefore, the injection timing can be controlled by adopting the flow rate in the resistant channel by adjusting the resistance such as the diameter or length of the channel. The flow rate can be calculated with below equation.

$$Q = P/R = \left[\rho\omega^2\left(R_{outer} - R_{inner}\right)\left(\frac{R_{outer} + R_{inner}}{2}\right)\right]/\left[16\eta L/A{d_H}^2\right]$$

where ρ denotes the working liquid density, ω denotes the angular velocity, A and $d_H$denote the cross-sectional area and equivalent diameter ($= 4HW/(H + W)$) of the microchannel, respectively, and R_{outer} and R_{inner} denote the distance to the leading meniscus or upstream meniscus from the rotation center. The volume of the secondary reservoir was determined by a mount of reagent which is used.

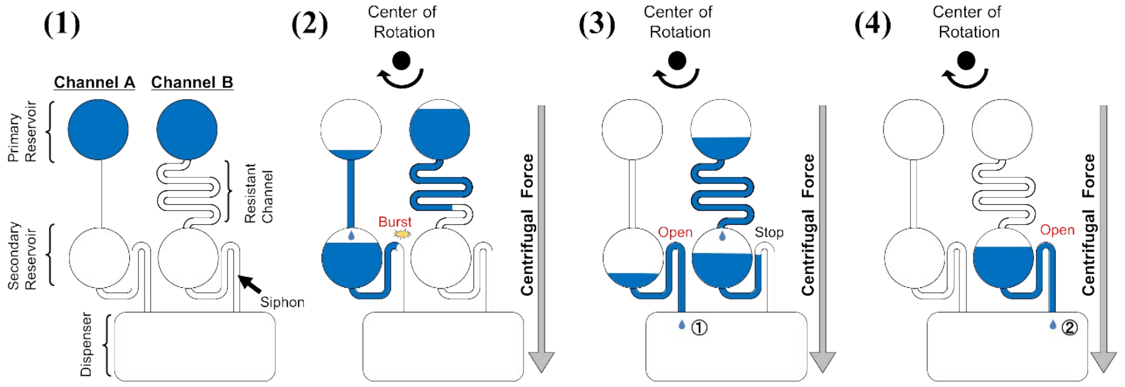

Fig. S2 Operation principle of CLOCK circuit

Device fabrication

We fabricated PDMS microchips for prototyping device in this paper. The fabrication method of multiplexed ELISA devise is shown below.

The microfluidic chips were fundamentally fabricated using general photolithography and soft lithography methods. First, microchannels and footprints of chambers were patterned on a 4-inch Si wafer by a photolithography as shown in Fig. S3(b). We used some ways to foam reservoirs and chambers. Reaction chambers and secondary reservoirs were formed the shape on the photoresist (Fig. (c)), and the shapes were transferred to PDMS chips by soft lithography as shown in Fig. (d). Reaction chamber's shape was formed with a reflow process using wax, and secondary reservoir's shape was formed by mounting cut PMMA parts with a double side tape. Other chambers such as primary reservoirs, sample/standard inlet, and waste chambers were formed by punching out the PDMS chip after the soft lithography as shown in Fig. (e). Open face of Microchannels, each chamber and reservoir were closed by bonding a flat PDMS sheet as shown in Fig. (f). Then, the chip was set onto a CD substrate, and the surface of chips (without vent holes and the insert of micro pipette) were covered with clear adhesive tapes as shown in Fig. (g).

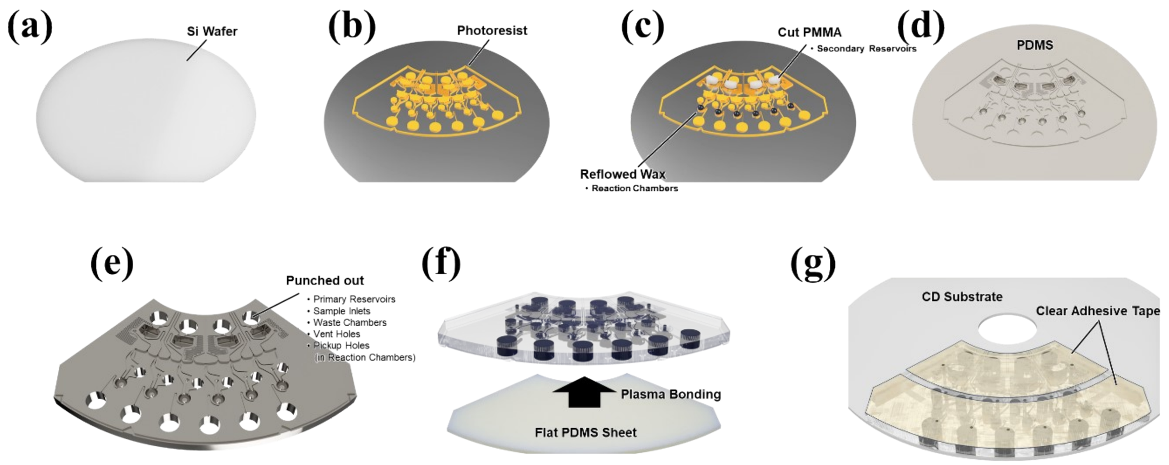

Fig. S3 Schematic of chip fabrication process
